# Hydroxysafflor Yellow A Inhibits LPS-Induced NLRP3 Inflammasome Activation via Binding to Xanthine Oxidase in Mouse RAW264.7 Macrophages

**DOI:** 10.1155/2016/8172706

**Published:** 2016-06-28

**Authors:** Xiaolong Xu, Yuhong Guo, Jingxia Zhao, Ning Wang, Junying Ding, Qingquan Liu

**Affiliations:** ^1^Beijing Hospital of Traditional Chinese Medicine, Capital Medical University, Beijing 100010, China; ^2^Beijing Institute of Traditional Chinese Medicine, Beijing 100010, China; ^3^Beijing Key Laboratory of Basic Research with Traditional Chinese Medicine on Infectious Diseases, Beijing 100010, China

## Abstract

Hydroxysafflor yellow A (HSYA) is an effective therapeutic agent for inflammatory diseases and autoimmune disorders; however, its regulatory effect on NLRP3 inflammasome activation in macrophages has not been investigated. In this study, we predicted the potential interaction between HSYA and xanthine oxidase (XO) via PharmMapper inverse docking and confirmed the binding inhibition via inhibitory test (IC_50_ = 40.04 *μ*M). Computation docking illustrated that, in this HSYA-XO complex, HSYA was surrounded by Leu 648, Leu 712, His 875, Leu 873, Ser 876, Glu 879, Phe 649, and Asn 650 with a binding energy of −5.77 kcal/M and formed hydrogen bonds with the hydroxyl groups of HSYA at Glu 879, Asn 650, and His 875. We then found that HSYA significantly decreased the activity of XO in RAW264.7 macrophages and suppressed LPS-induced ROS generation. Moreover, we proved that HSYA markedly inhibited LPS-induced cleaved caspase-1 activation via suppressing the sensitization of NLRP3 inflammasome and prevented the mature IL-1*β* formation from pro-IL-1*β* form. These findings suggest that XO may be a potential target of HSYA via direct binding inhibition and the combination of HSYA-XO suppresses LPS-induced ROS generation, contributing to the depression of NLRP3 inflammasome and inhibition of IL-1*β* secretion in macrophages.

## 1. Introduction

Inflammation plays a key role in innate immune responses during infections with invading pathogens such as bacteria, fungus, virus, and parasite [1]. Inflammation can be protective by recruiting immune and inflammatory cells and eliminating pathogens; however, excessive inflammation with overexpression of inflammatory factors and cytokines commonly leads to kinds of autoimmune diseases and host tissue injury, such as inflammatory bowel disease (IBD), psoriasis, and sepsis [2–4].

Pattern-recognition receptors (PRRs) are essential to regulate immune responses by targeting pathogenic microbes and presenting antigens to the adaptive immune system [5]. PRRs can be divided into four categories: toll-like receptors (TLRs), nucleotide-binding domain leucine-rich repeats (NLRs), nucleotide-binding oligomerization domain (NOD), and retinoic acid-inducible gene I-like receptors (RLRs) [6]. In the presence of microbial stimuli, host PRRs such as TLRs promote nuclear factor-kappa B (NF-*κ*B) mediated proinflammatory cytokines expression such as interleukin- (IL-) 1*β*, IL-6, tumor necrosis factor- (TNF-) *α*, and nitric oxide (NO) [7]. Meanwhile, another group of PRRs including NLR family pyrin domain-containing 3 (NLRP3) recruit the adaptor protein apoptosis-associated speck-like protein containing CARD (ASC) and procaspase-1. Formation of this complex leads to the activation of caspase-1 by triggering procaspase-1 self-cleavage and promotes the precursor forms of cytokines such as pro-IL-1*β* and pro-IL-18 into active forms that are secreted [8]. Activation of macrophages triggered by PRRs further induces the maturation of dendritic cells (DCs) by releasing proinflammatory cytokines and promotes the induction of adaptive immune responses [9].

The NLRP3 inflammasome is mainly expressed in immune and inflammatory cells such as macrophages, monocytes, neutrophils, and DCs when challenged by microbial stimuli. It has been well-established that multiple pathways participate in inflammasome activation such as cellular and mitochondrial reactive oxygen species (ROS), cathepsin B, and cytosolic protein kinase R (PKR) [10–12]. Among all of these triggers, ROS generation is reported to play an essential role in the activation of NLRP3 inflammasome when challenged by stimuli such as lipopolysaccharides (LPS), adenosine triphosphate (ATP), and urate crystals. More fundamentally, a more recent study demonstrated that ROS derived by XO, the oxidized form of xanthine dehydrogenase, is the main source of NLRP3 activation in macrophages, leading to excessive IL-1*β* and IL-18 secretion [13]. Inhibition of XO by Febuxostat, well-documented XO inhibitor, could significantly decrease the ROS generation and IL-1*β* secretion in macrophages stimulated by LPS, indicating the predominant role of XO in LPS-induced IL-1*β* mature and secretion [14, 15].

**Figure 1:**
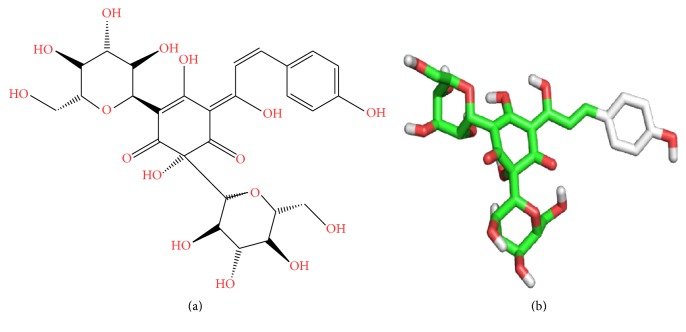
The 2D and 3D structure of HSYA.

HSYA (2D and 3D structure in Figure 1) is a water soluble monomer extracted from* Carthamus tinctorius* L. (Safflower), which has long been used for treatment of cardiovascular diseases in traditional Chinese medicine [16]. Recent researches showed that, besides the therapeutic effects upon cardiovascular system, HSYA exhibits promising anti-inflammatory properties by suppressing innate immune TLR4-inducing pathway, bettering LPS-induced inflammatory injury, scavenging excessive ROS, and inhibiting proinflammatory cytokines generation [17–19]. However, few studies have attempted to uncover the direct target of HSYA and interpret the mechanisms of its anti-inflammatory properties. In this study, we tried to find the potential target of HSYA via inverse prediction method and computation docking and further assessed the role of HSYA in regulating NLRP3/caspase-1/IL-1*β* pathway in macrophages.

## 2. Materials and Methods

### 2.1. Reagents

HSYA [>98% high-performance liquid chromatography (HPLC) purity] was purchased from Tauto Biotech (Shanghai, China). LPS (*Escherichia coli* O55:B5) was purchased from Sigma-Aldrich Chemical (St. Louis, MO, USA). Fetal bovine serum (FBS), Dulbecco's modified Eagle medium (DMEM), antibiotic-antimycotic, and TRIzol® reagents were purchased from Gibco (Grand Island, NY, USA). Bicinchoninic acid (BCA) protein assay kit was purchased from Pierce (Rockford, IL, USA). Enzyme-linked immunosorbent assay (ELISA) kits for mouse IL-1*β* and IL-18 were purchased from Cusabio (Wuhan, China). Antibodies for mouse *β*-actin, GAPDH, pro-IL-1*β*, IL-1*β*, NLRP3, ASC, procaspase-1, and caspase-1 were purchased from Cell Signaling Technology (Danvers, MA, USA). Antibody for XO was purchased from Santa Cruz (Dallas, TX, USA). The goat anti-mouse antibody was purchased from LI-COR Odyssey® (Lincoln, NE, USA). Probe DCFH_2_DA was purchased from Invitrogen (Carlsbad, CA, USA). XO activity assay kit (C/F) was purchased from BioVision (Milpitas, CA, USA). XO active protein was purchased from Abcam (Cambridge, MA, USA).

### 2.2. Cell Culture

RAW264.7 macrophage cell line was purchased from the American Type Culture Collection (Rockville, MD, USA). Cells were cultured in DMEM supplemented with 10% FBS and antibiotics (100 U/mL penicillin and 100 U/mL streptomycin) at 37°C in a humidified incubator under 5% CO_2_.

### 2.3. Enzyme-Linked Immunosorbent Assay

To detect the levels of cytokines (IL-1*β* and IL-18) released by RAW264.7 macrophages* in vitro*, cells were preincubated in 24-well plates (4 × 10^5^ cells/well) for 12 h and pretreated by HSYA (25, 50, and 100 *μ*M) for 3 h. Cells were then washed twice with PBS and challenged by LPS (1 *μ*g/mL) for another 24 h at 37°C under 5% CO_2_. The supernatants were collected and assayed immediately using sandwich technique. All protocols were performed following the manufacturer's instructions.

### 2.4. Western Blot Analysis

Cells (1 × 10^6^) were preincubated at 37°C under 5% CO_2_ for 12 h and then treated by appropriate experimental reagents. Cells were then harvested on ice, washed three times with cold PBS, and suspended in 500 *μ*L lysis buffer supplemented with protease inhibitors. After incubation on ice for 30 min, cell extracts were centrifuged at 14,000 rpm for 15 min at 4°C to isolate total cell proteins, which were quantified using a BCA protein assay kit. Proteins were separated by SDS-PAGE and electrotransferred to nitrocellulose membranes (Pierce, IL, USA) before hybridization with the appropriate detection antibodies (XO, pro-IL-1*β*, IL-1*β*, NLRP3, ASC, procaspase-1, and caspase-1). GAPDH was used to correct for differences in loading of the proteins and for normalization of the densitometric values of immunoblot signals obtained from three separate experiments using LI-COR Odyssey detecting system.

### 2.5. Quantitative RT-PCR Analysis

Macrophages were preincubated in 6-well plates (1 × 10^6^ cells/well) for 12 h and pretreated by HSYA (25, 50, and 100 *μ*M) 3 h prior to LPS (1 *μ*g/mL) stimulation. Total RNA was extracted using TRIzol reagent. The concentration and integrity of total RNA samples were evaluated by measurement of the* A*260/280 ratio. Quantitative polymerase chain reaction (PCR) analysis was performed using the DNA Engine Mx3000P® (Agilent, Santa Clara, CA, USA) fluorescence detection system according to optimized PCR protocols. The following primers were used for amplification: *β*-actin: F: 5′-CCCATCTACGAGGGCTATGC-3′, R: 5′-GGTGTAAAACGCAGCTCAGTA-3′; XO, F: 5′-CACGATGACGAGGACAACGG-3′, R: 5′-TAGGCTCAGGCTTGTTTCGG-3′.

### 2.6. Measurement of ROS Production

ROS production was detected by measuring intracellular ROS formation using probe DCFH_2_DA. Briefly, RAW264.7 cells were pretreated with HSYA (100 *μ*M) or positive control Febuxostat (30 *μ*M) for 3 h and then stimulated with LPS (1 *μ*g/mL) for 6 h to promote ROS generation. Cells were washed twice with PBS and then incubated with probe DCFH_2_DA (20 nM) for 15 min. Fluorescence staining was visualized using a fluorescence microscopy (Olympus, IX71) and fluorescence assays were measured with a fluorescence microplate reader (Tecan, Sunrise) at excitation/emission 525/610 nm.

### 2.7. Xanthine Oxidase Activity Detection

Theoretically, XO oxidizes xanthine to hydrogen peroxide (H_2_O_2_) which reacts stoichiometrically with OxiRed*™* Probe to generate color at *λ* = 570 nm and fluorescence at *E*_*x*_/*E*_*m*_ = 535/587 nm. Since the color or fluorescence intensity is proportional to XO content, the XO activity can be accurately measured.

To detect the direct inhibition of HSYA on XO, we first added HSYA at various concentrations (0, 10, 20, 40, 80, 160, 320, 640, and 1280 *μ*M) into active XO solution and incubated at 37°C for 30 min. After that, this mixed solution was added into a reaction system that consisted of assay buffer, substrate mix, enzyme mix, and OxiRed probe and incubated for another 30 min at 37°C. The plate was then measured immediately at *E*_*x*_/*E*_*m*_ = 535/587 nm. The IC_50_ of HSYA on XO was calculated by SPSS Probit regression.

To detect the effect of HSYA on XO activity in LPS-stimulated RAW264.7 macrophages, we treated cells with HSYA at different concentrations (25, 50, and 100 *μ*M) for 3 h before LPS challenge (1 *μ*g/mL, 12 h). Febuxostat (30 *μ*M) was employed as positive control. Clear XO extract was obtained by centrifuge (16,000 ×g, 10 min) and the activity of XO from cell extract was added into reaction system and measured as described above.

### 2.8. Pharmacophore Mapping Prediction of Potential Targets

PharmMapper server is a web server for potential drug targets identification using pharmacophore mapping approach at http://59.78.96.61/pharmmapper/. Briefly, 2D Mol2 file of HSYA (PubChem CID: 6443665) was submitted to PharmMapper server. During the procedure, the maximum conformations were set up to 300, and the number of reserved matched targets was 300. Other parameters were kept as default. The submission ID can be stored and used to check the prediction results.

### 2.9. Molecular Docking

Further, to characterize the combination between HSYA and XO, we used Autodock and AutoGrid to calculate the binding affinity and binding sites. Briefly, the two-dimensional (2D) structure of HSYA was drawn and converted to Mol2 format by Chemdraw. Then the Mol2 file of HSYA was optimized and saved as final coordinate pdb file for docking study. The 3D crystal structure of XO (entry code 1F04) was downloaded from the Protein Data Bank (PDB) at http://www.rcsb.org/. The grid procedure was handled, by AutoGrid, in a grid box of 60*∗*60*∗*60 Å with a 1.0 Å to enclose all the active sites and to allow for the flexible rotations of HSYA. Then, the computation was performed 30 times using Autodock 4.0 and results were saved.

### 2.10. Statistical Analysis

The data were presented as means ± standard error of the mean (SEM) and differences between mean values of normally distributed data were assessed by the one-way analysis of variance (ANOVA) followed by Duncan's test for multiple comparisons. Differences were considered significant at *P* ≤ 0.05.

## 3. Results

### 3.1. PharmMapper Inverse Docking for Potential Target of HSYA

**Figure 2:**
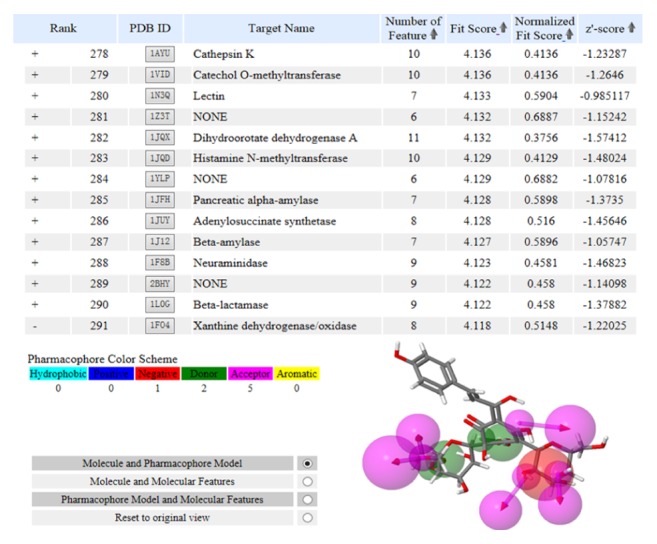
XO is a potential target of HSYA via PharmMapper inverse docking. 300 potential candidates out of 7302 were listed and sorted according to the fit score. The molecule and pharmacophore model of XO (PDB ID: 1F04) was exhibited right below the selected target. Note: pharmacophore features are schemed by color: hydrophobic, cyan; positive, blue; negative, red; donor, green; acceptor, magenta.

Via pharmacophore mapping approach, 300 potential candidates out of 7302 were listed and sorted according to the fit score (Submission ID 151015052728). Based on the disease information and potential roles in inflammation and redox related signaling pathways, endothelial nitric oxide synthase (PDB ID: 1P6M), NADPH cytochrome P450 reductase (PDB ID: 1J9Z), and XO (PDB ID: 1F04) were selected as potential targets of HSYA. Based on the pharmacophore model, endothelial nitric oxide synthase had one hydrophobic, four donors, and three acceptors; NADPH cytochrome P450 reductase had two hydrophobics, one positive, two negatives, one donor, eight acceptors, and one aromatic; XO had one negative, two donors, and five acceptors. However, since inhibition tests showed that HSYA possessed no depression effects on activities of endothelial nitric oxide synthase and NADPH cytochrome P450 reductase (data not shown), only the pharmacophore model of XO was exhibited in Figure 2.

### 3.2. HSYA Inhibits the Activity of XO via Direct Combination

**Figure 3:**
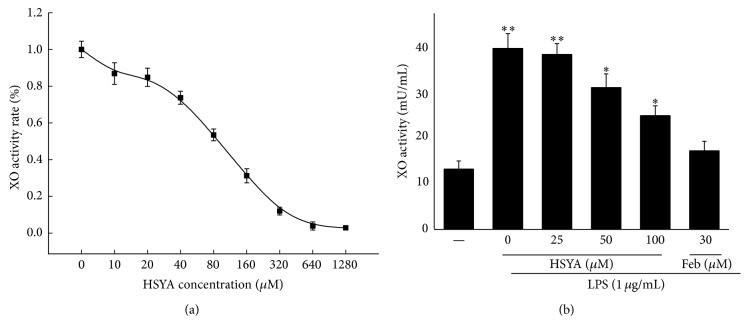
HSYA inhibits XO activity both out of cells and in cells. (a) HSYA at various concentrations (0, 10, 20, 40, 80, 160, 320, 640, and 1280 *μ*M) was incubated with active XO at 37°C for 30 min. Mixed solutions were then added into reaction system and the reads were measured by fluorescence detection. The rate was calculated as the ratio of certain concentration/negative control (0 *μ*M HSYA). (b) RAW264.7 macrophages were pretreated by HSYA (25, 50, and 100 *μ*M) and Febuxostat (30 *μ*M) for 3 h and then challenged by 1 *μ*g/mL LPS for another 12 h. The activity of XO from cell extract was measured by fluorescence detection. Data represent the mean ± SEM of three independent experiments and differences between mean values were assessed by one-way ANOVA. ^*∗*^ *P* < 0.05 and ^*∗∗*^ *P* < 0.01 indicate significant difference compared with the control group.

**Figure 4:**
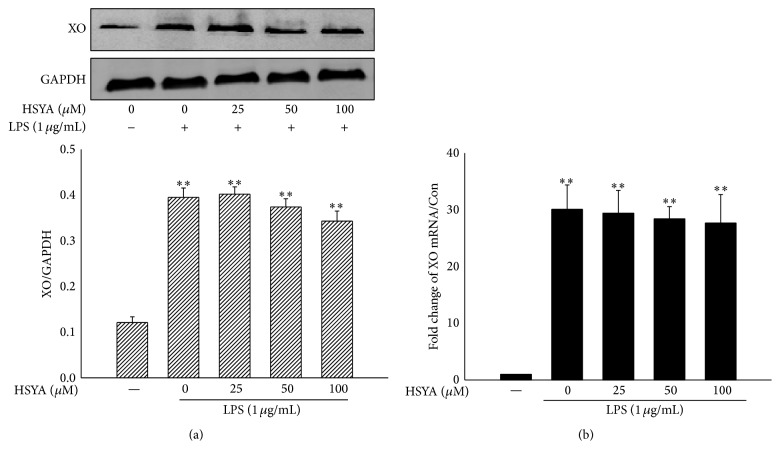
HSYA possesses no regulatory effect on XO expression. (a) RAW264.7 macrophages were pretreated by HSYA (25, 50, and 100 *μ*M) for 3 h and then challenged by 1 *μ*g/mL LPS for another 8 h. The XO protein expression was analyzed by Western Blot. (b) RAW264.7 macrophages were pretreated by HSYA (25, 50, and 100 *μ*M) for 3 h and then challenged by 1 *μ*g/mL LPS for another 6 h. The XO mRNA expression was analyzed by RT-PCR. Data represent the mean ± SEM of three independent experiments. ^*∗∗*^ *P* < 0.01 indicates significant difference compared with the control group.

To confirm if there is a direct interaction between HSYA and XO, we detected the inhibitory effect of HSYA on XO activity both out of cells and within cells. Febuxostat was used as positive control. Because HSYA liquor appears yellow, we employed fluorescence detection, other than OD detection, to reduce error. Results showed that HSYA exhibited remarkable inhibitory effect on XO activity with IC_50_ = 40.04 *μ*M out of cells (Figure 3(a)). The IC_50_ of positive control Febuxostat was 3.24 *μ*M (data not shown). In addition, as shown in Figure 3(b), 50–100 *μ*M HSYA treatment started to exhibit suppression on the activity of XO in LPS-stimulated macrophages (*P* < 0.05). However, results demonstrated that XO treatment had no noticeable action on the protein and mRNA expression of XO in macrophages (*P* > 0.05) (Figure 4).

### 3.3. Molecular Docking Studies

**Figure 5:**
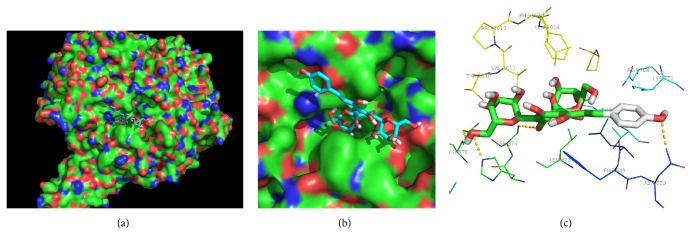
Binding pattern of HSYA-XO complex. Autodock and AutoGrid were used to calculate the binding affinity. The grid procedure was handled by AutoGrid, in a grid box of 60*∗*60*∗*60 Å with a 1.0 Å to enclose all the active sites and to allow for the flexible rotations of HSYA. Then, the computation was performed 30 times using Autodock 4.0. (a) Overview. (b) Amplified binding pattern. (c) 3D interaction diagram between HSYA and XO. HSYA: green; hydrogen bond: yellow dash line.

Since we confirmed that HSYA inhibits the activity of XO, we tried to further understand the interaction between HSYA and XO by computation docking. From the molecular docking data, we concluded HSYA directly implanted into the activity pocket of XO by hydrophobic characteristic and hydrogen bonds, which are essential to this interacting system with a binding energy of −5.77 kcal/M (Table 1). In this HSYA-XO complex, HSYA was surrounded by Leu 648, Leu 712, His 875, Leu 873, Ser 876, Glu 879, Phe 649, and Asn 650 in hydrophobic interactions (Figures 5(a) and 5(b)). Moreover, Glu 879, Asn 650, and His 875 were observed to form hydrogen bonds with the hydroxyl groups of HSYA (Figure 5(c)).

### 3.4. HSYA Inhibited ROS Generation in Macrophages

**Figure 6:**
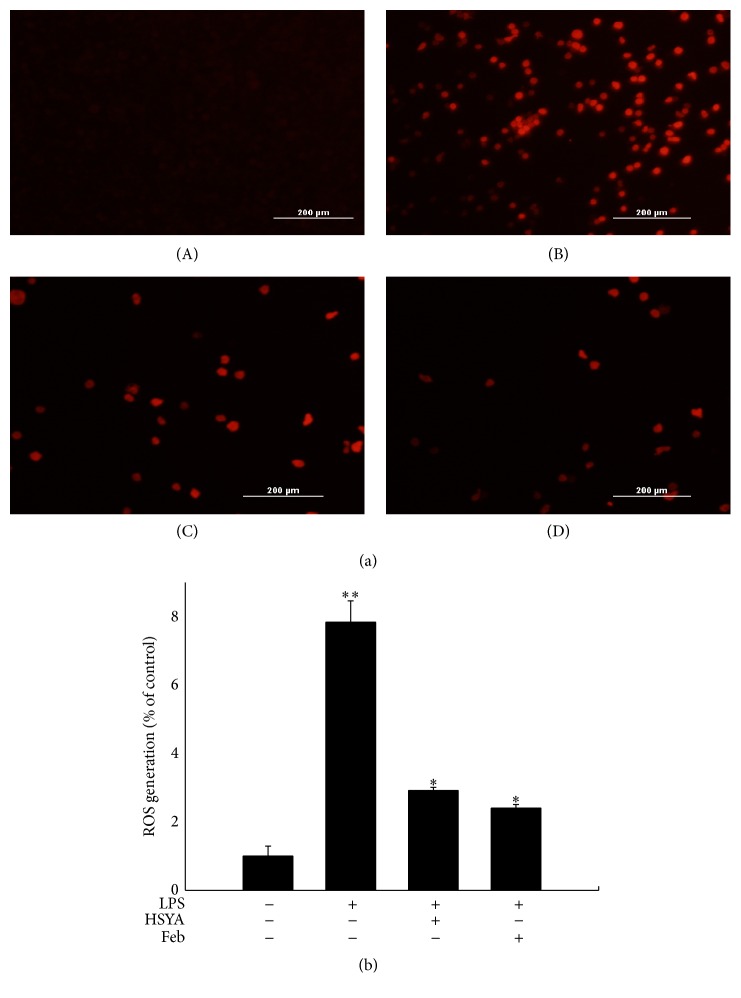
HSYA inhibits LPS-induced excessive ROS generation in RAW264.7 macrophages. (a) ROS detection was performed using a fluorescence macroscopy. (A) RAW264.7 cells were cultured in DMEM for 6 h and then incubated with probe DCFH_2_DA for 15 min. (B) RAW264.7 cells were treated with 1 *μ*g/mL LPS for 6 h and then incubated with probe DCFH_2_DA for 15 min. (C) RAW264.7 cells were pretreated with 100 *μ*M HSYA for 3 h and treated with 1 *μ*g/mL LPS for 6 h and then incubated with probe DCFH_2_DA for 15 min. (D) RAW264.7 cells were pretreated with 30 *μ*M Febuxostat for 3 h and treated with 1 *μ*g/mL LPS for 6 h and then incubated with probe DCFH_2_DA for 15 min. (b) ROS generation was measured by fluorescence microplate reader. Data represent the mean ± SEM of three independent experiments and differences between mean values were assessed by one-way ANOVA. ^*∗*^ *P* < 0.05 and ^*∗∗*^ *P* < 0.01 indicate significant differences compared with the control group.

As a main source of ROS, XO in macrophages can be activated by LPS and leads to excessive ROS generation. To investigate if XO inhibition by HSYA results in less ROS production, we evaluated the ROS level via fluorescence detection. As shown in Figure 6, LPS challenge markedly increased the amount of ROS in macrophages (*P* < 0.01); however, 100 *μ*M HSYA pretreatment for 3 h significantly suppressed LPS-induced ROS generation (*P* < 0.05), with no remarkable difference compared with 30 *μ*M positive control Febuxostat (*P* > 0.05).

### 3.5. HSYA Suppresses the Activation of NLRP3 Inflammasome

**Figure 7:**
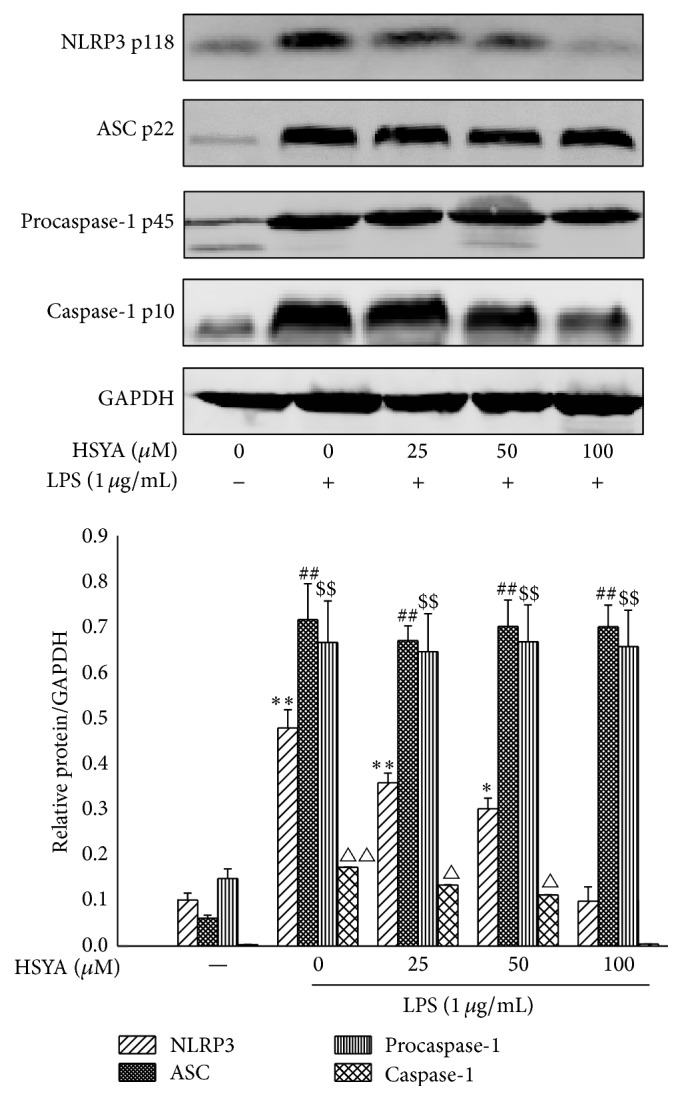
HSYA inhibits NLRP3 inflammasome activation. RAW264.7 cells were pretreated with HSYA at indicated doses (25, 50, and 100 *μ*M) for 3 h and then treated with 1 *μ*g/mL LPS for 8 h. The GAPDH, NLRP3, ASC, procaspase-1, and caspase-1 proteins expressions were analyzed by Western Blotting. Data represent the mean ± SEM of three independent experiments and differences between mean values were assessed by one-way ANOVA. ^*∗*^ *P* < 0.05, ^*∗∗*^ *P* < 0.01, ^$$^ *P* < 0.01, ^##^ *P* < 0.01, ^△^ *P* < 0.05, and ^△△^ *P* < 0.01 indicate significant differences compared with the control group of indicated proteins, respectively.

Since latest research demonstrated that activation of NLRP3 inflammasome requires the ROS generated by XO, we further detected the effect of HSYA on activation of NLRP3 inflammasome. As shown in Figure 7, 1 *μ*g/mL LPS challenge significantly enhanced the expression of NLRP3, ASC, and procaspase-1 proteins (*P* < 0.01), indicating that NLRP3 inflammasome was activated by LPS in macrophages. HSYA treatment from 50 to 100 *μ*M notably inhibited the expression of NLRP3 (*P* < 0.05); however, no significant regulatory effects were observed on expression of ASC and procaspase-1 (*P* > 0.05). NLRP3 is essential to activate caspase-1 by cleaving procaspase-1 into mature form. Hence, we detected the effect of HSYA on cleaved caspase-1 expression. Result showed that HSYA treatment from 50–100 *μ*M exhibited remarked inhibitory effect on the mature caspase-1 expression in macrophages (*P* < 0.05).

### 3.6. HSYA Inhibits the Expression of IL-1*β* and IL-18 in Macrophages

**Figure 8:**
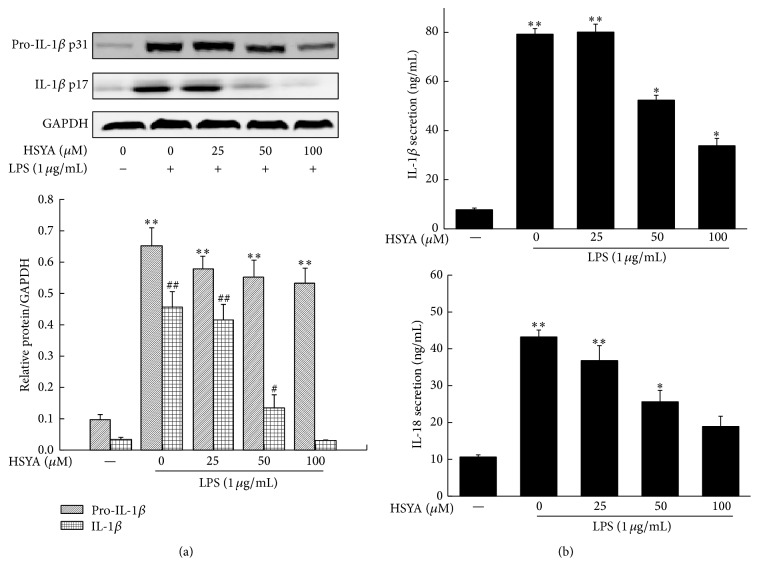
HSYA inhibits LPS-induced IL-1*β* and IL-18 secretion. RAW264.7 cells were pretreated with HSYA at indicated doses (25, 50, and 100 *μ*M) for 3 h and then treated with 1 *μ*g/mL LPS for 12 h (Western Blot) or 24 h (ELISA). (a) The pro-IL-1*β*, IL-1*β*, and GAPDH proteins expressions were analyzed by Western Blotting. (b) The IL-1*β* and IL-18 secretion were measured by ELISA. Data represent the mean ± SEM of three independent experiments and differences between mean values were assessed by one-way ANOVA. ^*∗*^ *P* < 0.05, ^*∗∗*^ *P* < 0.01, ^#^ *P* < 0.05, and ^##^ *P* < 0.01 indicate significant differences compared with the control group of indicated proteins, respectively.

To further estimate the inhibition of HSYA on NLRP3 inflammasome activation, we checked the expression of NLRP3 downstream cytokines IL-1*β* and IL-18. As shown in Figure 8(a), HSYA treatment at concentrations from 50 to 100 *μ*M notably decreased the protein expression of IL-1*β* in a dose-dependent manner (*P* < 0.05). Results based on Western Blot also displayed that 100 *μ*M HSYA possessed an observable negative regulatory effect on pro-IL-1*β* expression, but with no significant difference compared with LPS group. Moreover, data in Figure 8(b) showed that, as expected, HSYA treatment at concentrations from 50 to 100 *μ*M markedly decreased the secretion level of IL-1*β* and IL-18 in supernatant (*P* < 0.05).

## 4. Discussion

XO is one of the major enzymatic sources of ROS by utilizing molecular oxygen as a substrate to break down xanthine and hypoxanthine into O_2_^∙−^ and hydrogen peroxide [20]. It has been well-documented that the activity of XO can be enhanced by kinds of stimuli including LPS and cytokines in macrophages, resulting in many pathologic conditions characterized by oxidative stress and inflammation [21, 22]. Inhibition of XO by pharmacological inhibitors such as Febuxostat and allopurinol markedly decreases XO-induced excessive ROS generation and betters the pathologic conditions, suggesting the essential role of XO in inflammation and providing potential strategy of therapies to cure inflammation diseases [14, 23, 24]. By employing PharmMapper, a server computationally predicting candidates of submitted small molecule, drug, and compounds using inverse docking technique [25], we found that endothelial nitric oxide synthase, NADPH cytochrome P450 reductase, and XO may be potential disease-related targets of HSYA. However, data from subsequent inhibitory tests showed that HSYA only possessed promising regulatory effect on XO instead of endothelial nitric oxide synthase and NADPH cytochrome P450 reductase. The pharmacophore outcome disposed to the molecule characteristics of HSYA, which convincingly supports the inverse docking result provided by PharmMapper. To better understand the chemical-protein combination information of HSYA and XO, we employed Autodock and AutoGrid to investigate the interaction of HSYA-XO complex. Results demonstrated that HSYA embedded into the active region of XO via hydrophobic interaction, a critical binding force for complex stabilization [26], with many amino acids including Leu 648, Leu 712, His 875, Leu 873, Ser 876, Glu 879, Phe 649, and Asn 650. Besides, the hydrogen bond interactions between HSYA and XO at residues such as Glu 879, Asn 650, and His 875 also contribute to the combination and endow the inhibitory effect of HSYA on XO activity. Since we proved that HSYA directly inhibits the activity of XO, we tried to further investigate whether HSYA affects the expression of XO and leads to changes in downstream signaling pathways. However, results based on RT-PCR and Western Blot demonstrated that HSYA possesses limited regulatory effect on LPS-induced overexpression of XO at mRNA and protein level, suggesting that HSYA exhibits subsequent regulating properties through inhibiting the activity but not amount of XO in LPS-challenged macrophages.

ROS overproduction triggered by phagocytes is critical for the cellular signaling cascades in macrophages during immune activation [27–29]. Since it has been well-documented that XO plays an important role in ROS generation, we then detected the effect of HSYA on LPS-induced ROS production in RAW264.7 macrophages. Results indicated that 100 *μ*M HSYA pretreatment notably suppressed LPS-induced excessive ROS generation, with no significant difference compared with 30 *μ*M XO inhibitor Febuxostat. Knowing that HSYA owns considerable antioxidant property [30, 31], our findings may partially uncover the mechanism of which HSYA decreases ROS generation. Since it has been proved that XO-induced ROS generation is the dominant trigger of NLRP3 inflammasome [13], we further investigated the effect of HSYA on NLRP3 inflammasome activation.

NLR family, well-established PRR, is critical for promoting inflammation process in host innate immune response when challenged by infectious stimuli [32]. Among these inflammasomes, NLRP3 has been characterized in most mammalian cells. Studies in macrophages and various animal models demonstrated that the activation of NLRP3 inflammasome can be observed in kinds of autoimmune and inflammatory diseases such as experimental autoimmune encephalomyelitis (EAE) [33], multiple sclerosis [34], inflammatory bowel disease including ulcerative colitis, and Crohn's disease [35, 36]. Moreover, NLRP3 inflammasome activation may contribute to insulin resistance and type II diabetes [37], uric acid accumulation, and gout [38]. Mechanically, when stimulated by PRRs, NLRP3 inflammasome activates and then promotes the recruitment of ASC and the procaspase-1, resulting in the formation of caspase-1 p10 fragment which contributes to active caspase-1 enzyme that cleaves pro-IL-1*β* into mature IL-1*β* form. Results based on Western Blot illustrated that HSYA treatment notably decreased the cleaved caspase-1 expression, indicating that HSYA could suppress LPS-induced activation of NLRP3 inflammasome. To better understand the regulatory effect of HSYA on NLRP3 inflammasome, we detected the protein expression of NLRP3, ASC, and procaspase-1 complex. Results revealed that HSYA notably inhibited LPS-induced NLRP3 expression; however, no noticeable suppressions were observed on the upregulated expression of ASC and procaspase-1. A more recent study in human and mice macrophages demonstrated that ROS generation caused by XO, instead of NOX, is the major source that promotes NLRP3 inflammasome activation [13]. Taken together, we conferred that HSYA treatment restrained the activation of NLRP3 inflammasome via directly binding to XO and inhibiting the ROS overproduction.

IL-1*β* and IL-18 are two well-documented proinflammatory cytokines that play central role in inflammation and autoinflammatory disorders. It has been established that secretion of IL-1*β* and IL-18 needs the participation of NLRP3 inflammasome [39, 40]. Hence, we measured the protein expression of pro-IL-1*β* and IL-1*β* in macrophages and the secretion of IL-1*β* and IL-18 in the supernatant. As expected, HSYA pretreatment notably decreased LPS-induced cleaved IL-1*β* expression, indicating that HSYA inhibited the cleaving process of IL-1*β* mainly by suppressing the NLRP3 inflammasome activation. Meanwhile, mild abatement of pro-IL-1*β* expression was observed under HSYA pretreatment. Considering the important role of ROS generation in the activation of NF-*κ*B [41], a classical nuclear transcription factor that encodes kinds of proinflammatory cytokines including IL-1*β*, we speculated that ROS scavenging by HSYA partially depressed the activation of NF-*κ*B and led to lower pro-IL-1*β* protein expression. However, the inhibition of NLRP3 eventually blocked the transformation process from pro-IL-1*β* to cleaved IL-1*β*. Detection on secretion of IL-1*β* and IL-18 by ELISA further proved that HSYA inhibited NLRP3 inflammasome activation and finally suppressed its downstream cytokines secretion. But, since we did not find the suitable antibodies for pro-IL-18 and IL-18 detection, we could not prove whether the downregulation of IL-18 was caused by blocking mature process.

## 5. Conclusion

**Figure 9:**
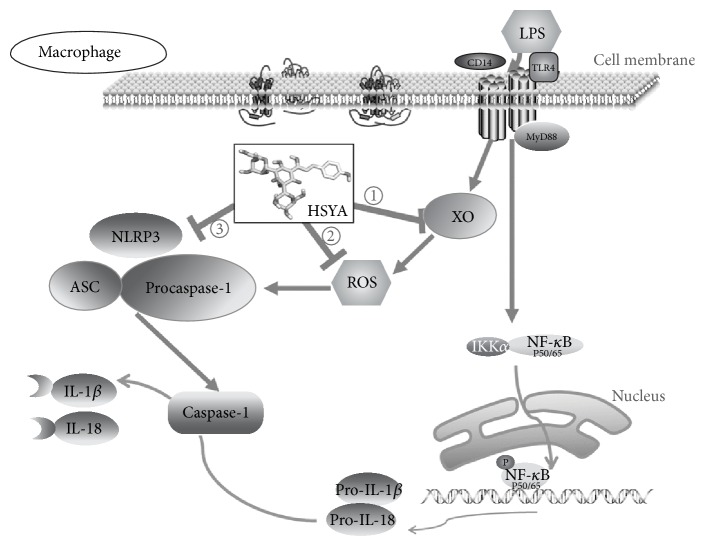
Proposed models demonstrating the mechanism that HSYA inhibits NLRP3 inflammasome activation via directly binding to XO in macrophages. ① HSYA first binds to XO and suppresses the activity of XO upregulated by LPS. ② HSYA then inhibits LPS-induced excessive ROS generation. ③ HSYA suppresses NLRP3 inflammasome activation and thus blocked the mature transformation process of IL-1*β* and IL-18.

Taken together, we found that XO is a potential target of HSYA using PharmMapper inverse docking and computer simulation. The inhibitory effect of HSYA on XO activity contributes to the ROS scavenging and NLRP3 inflammation suppression triggered by LPS, leading to decreased IL-1*β* and IL-18 secretion in RAW264.7 macrophages (Figure 9). These findings suggest a potential role of HSYA in pathogenesis of various inflammatory diseases.

## Figures and Tables

**Table 1:** The binding information of HSYA-XO complex.

Ligand	Name	Ligand length (Å)	Binding energy (kal/M)	Binding force	Binding mode
HSYA	XO	17.6	−5.77	Hydrophobic; hydrogen bonds; Van Edward force	Noncovalent bond
